# Proposing an avenue for suboptimal health research through the lens of tourism

**DOI:** 10.7189/jogh.12.03058

**Published:** 2022-09-06

**Authors:** Zheng Guo, Jun Wen, Danni Zheng, Zheng Yulu, Haifeng Hou, Wei Wang, Suboptimal Health Study Consortium (SHSC)

**Affiliations:** 1 Centre for Precision Health, Edith Cowan University, Joondalup, Australia; 2 School of Business and Law, Edith Cowan University, Joondalup, Australia; 3 Department of Tourism, Fudan University, Shanghai, China; 4 School of Public Health, Shandong First Medical University & Shandong Academy of Medical Sciences, Taian, China; 5 Beijing Key Laboratory of Clinical Epidemiology, School of Public Health, Capital Medical University, Beijing, China; 6 The First Affiliated Hospital of Shantou University Medical College, Shantou, Guangdong, China

The COVID-19 outbreak has posed tremendous threats to both global health and individuals’ psychological and physiological well-being. Scholars across the social and medical sciences are calling for multidisciplinary research regarding how the COVID-19 pandemic has affected global health [1]. As daily stressors continue to accumulate, the number of people reporting health complaints that cannot be detected by laboratory measures is on the rise [2,3]. These conditions can be complex and challenging to define but are generally deemed as “suboptimal health” [4]. Suboptimal health status (SHS) refers to a reversible state between health and illness [2]. It is characterized by health concerns (eg, back pain, headache, chronic fatigue) and constellations of symptoms (eg, anxiety, depression) that can affect one’s cardiovascular system [3,5,6], digestive system [7], immune system [4,8], and mental status [9,10]. Guidance from traditional Chinese medicine as reported by the China Association of Chinese Medicine suggests that SHS also hinders one’s adaptability, physiological state, and vitality [11].

SHS has gradually become a major public health concern worldwide. Its association with chronic diseases traverses multiple populations, such as in Japan, Canada, and Australia. For example, China’s urbanization has exploded in recent decades, with the urban population accounting for 17.9% of the total population in 1978 vs 58.5% in 2017 [12]. Risk factors for SHS can be classified into two categories: lifestyle behaviours and environmental factors [13]. Urbanization and accompanying environmental shifts have exacerbated SHS risk factors, including air pollution, noise, smoking, a sedentary lifestyle, a subpar diet, and work stress [14]. Although the molecular mechanism of SHS remains unclear, the condition is associated with changes in IgG N-glycosylation, telomerase length, metabolites, and hormone levels [4]. These metrics serve as objective and dynamic biomarkers in SHS assessment [8]. The current prevalence of SHS in China exceeds 65% [15], and it represents a serious concern in other countries as well [16]. Its prevalence may even be underreported. Many people do not realize they have the condition: among 6000 Chinese individuals who considered themselves “healthy”, 72.8% exhibited SHS [13].

**Figure:**
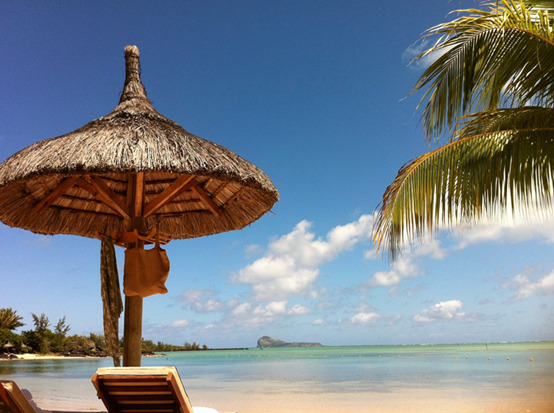
Photo: Tourism activities as potential interventions for SHS individuals. Source: Dr Danni Zheng, used with permission.

Amid increasing societal health concerns, especially as countries host larger ageing populations, the nexus between individuals’ health and tourism has been paid careful attention to in many disciplines. Tourism and hospitality represents one of the industries most affected by COVID-19 due to travel restrictions and social safety measures [17]. Interestingly, however, tourism’s role in public health has been largely neglected, despite documented benefits [18]. For instance, taking part in vacation activities has been shown to boost travellers’ physical and psychological well-being [19]. Leisure activities can also improve senior travellers’ well-being. The integration of physical and mental health has changed views on health promotion and disease prevention [20]. Well-being features pleasant moods, productivity, a lack of negative emotions, and a sense of fulfilment. Scholars have examined related aspects such as economic, emotional, physical, psychological, social, and workplace well-being, and life satisfaction. Yet despite SHS having become a public health issue, its relationship with tourism engagement has not been explored in either tourism or public health.

To date, no study has considered the potential relationship between tourism and SHS. Researchers also have yet to compare healthy tourists to those with SHS in hopes of improving global and public health. This viewpoint innovatively discusses the association between both topics and groups within a Chinese sample to shed light on certain tourism behaviours. Findings are intended to make meaningful contributions to the literature on both tourism and public health.

A cross-sectional study was conducted to explore the relationships among tourists’ characteristics and SHS between October and December 2021 in Shandong Province, China. Tourists’ demographic information and personal characteristics were measured using items drawn from prior research on tourism and suboptimal health. Demographic data included respondents’ gender, age, educational level, work stress, exercise strength, daily sleep duration, smoking behaviour, drinking behaviour, and monthly income. Tourist characteristics included one’s frequency of domestic travel or international travel, travel intensity, length of domestic travel or international travel, preference for domestic travel or international travel, and travel costs. SHS was assessed via the suboptimal health status questionnaire (SHSQ-25) [2], a validated tool of precision health care. This self-report instrument contains 25 items covering five dimensions: 1) fatigue, 2) the cardiovascular system, 3) the digestive tract, 4) the immune system, and 5) mental health. The SHSQ-25 questionnaire has been applied in studies of Chinese populations. Each item contains a statement scored on a 5-point Likert-type scale based on how often individuals have experienced uncomfortable symptoms in the preceding 3 months (0 = never or almost never, 1 = occasionally, 2 = often, 3 = very often, 4 = always). Respondents’ health status was stratified into two groups based on their total SHSQ-25 score: optimal health (OPH; total score <35) or SHS (total score ≥35) [3]. The final sample included 360 respondents. The prevalence of SHS was 36.39% (131/360) and was higher among women (46.07%; 88/191) than men (25.44%; 43/169).

These findings reveal statistically significant correlations both between respondents’ domestic travel frequency (tourism-related characteristic) and mental status (SHS domain) and between respondents’ domestic travel preferences (tourism-related characteristics) and mental status, cardiovascular system, and immune system (SHS domains). These results enrich the tourism and public health realms with respect to COVID-19. This study is one of the first to introduce SHS into tourism. Tourism activities may provide optimal intervention outcomes in leveraging SHS individuals’ strengths and well-being.

**Figure 1:**
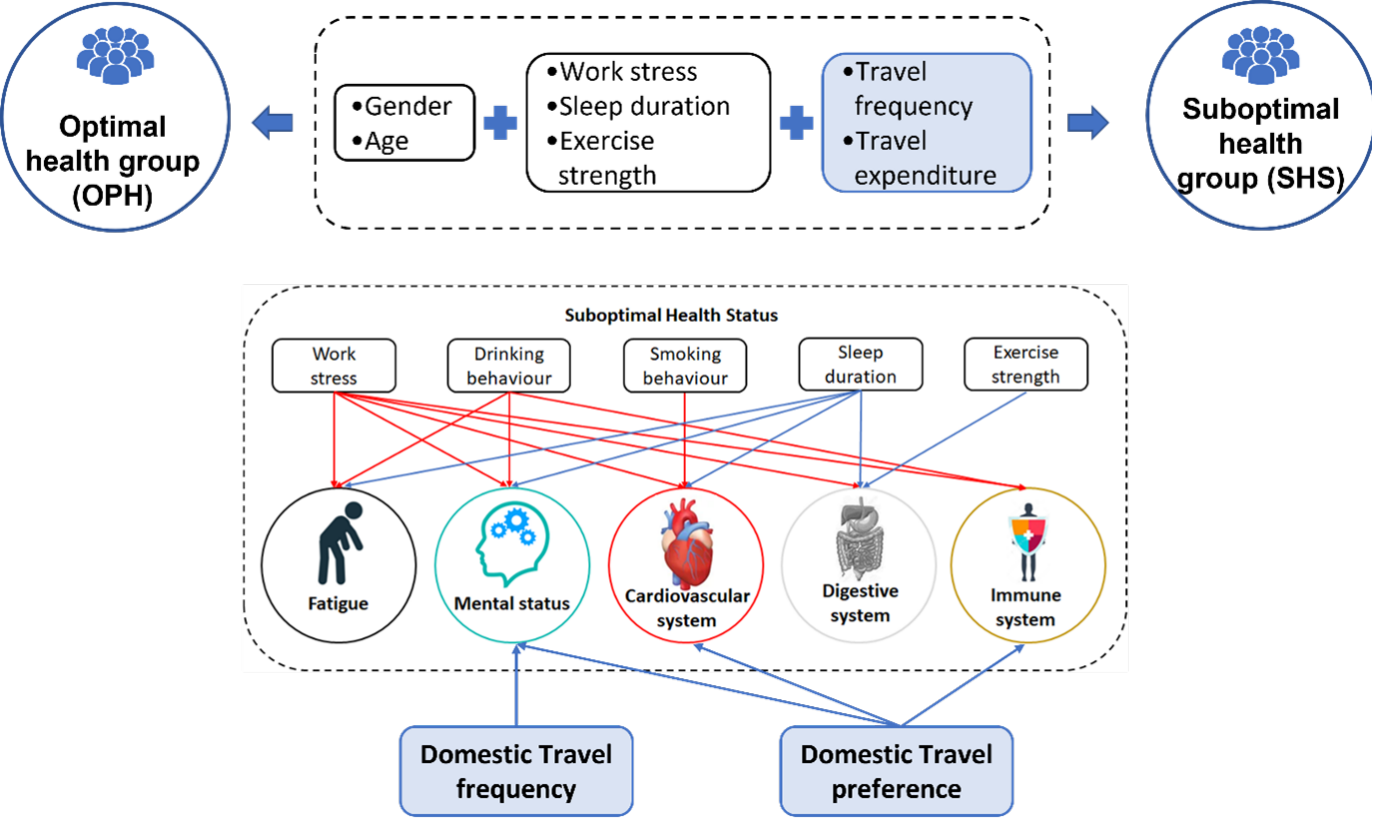
Significant relationships between demographic/tourist characteristics and SHS domains. Red lines indicate an increase in scores on specific SHS domains; blue lines indicate a decrease in scores on specific SHS domains.

As shown in **Figure 1**, several demographic factors (work stress, drinking, smoking, exercise strength, and monthly income) were each significantly associated with the SHS domains of fatigue, mental status, the cardiovascular system, the digestive system, and the immune system [2]. A healthy lifestyle, including little work-related stress, adequate sleep, limited alcohol consumption, and sufficient exercise, positively influenced Chinese tourists. Additionally, two tourism-related attributes were significantly correlated with specific SHS domains: tourists with SHS who engaged in domestic travel more frequently demonstrated better mental health. This correlation provides preliminary support for the role of tourism in individuals’ health. This finding also echoes prior studies citing tourism as a possible way to improve people’s mental health in general. Moreover, survey results indicated that tourists with SHS preferred to travel domestically and demonstrated fewer immune system problems. The sports medicine and immunology literature has highlighted a relationship between physical exercise and immune system development. Engaging in tourism activities requires a certain level of physical endurance. The current study’s results thus present initial evidence that tourism activities, especially among individuals with SHS, can improve their health. Because tourism-related activities (eg, camping, hiking, holidays, and religious travel) normally occur in nature or outdoor settings, cardiovascular disease risk can be lessened through greater physical activity. Tourism experiences also evoke mindfulness, happiness, and a sense of thriving. The psychological benefits of tourism therefore transcend exercise, the impact of which has been addressed in relation to tourists with mental health concerns. Positive psychology interventions are established clinical treatments. As a complement, tourism activities may promote productive treatment outcomes by capitalizing on the strengths and well-being of people with SHS.

This study applied an interdisciplinary approach to explore the nexus between tourism-related characteristics and SHS domains based on a Chinese sample. The preliminary findings, as discussed above, offer evidence-based insight for developing marketing strategies and tourism products to help travellers with SHS fully benefit from tourism. This study also serves as a springboard for subsequent work on the generally neglected segment of tourists with SHS. For instance, medical scientists can perform clinical trials to compare tourists with SHS and OHS. In the social sciences, qualitative interviews would enable scholars to gather information from travellers with SHS and from medical professionals familiar with the condition to discern these tourists’ trip preferences. Interdisciplinary approaches blending tourism, marketing, and medical science would allow for especially robust findings [1]. The effects of COVID-19 on individuals’ SHS warrant attention in and beyond tourism. Risk-taking behaviour is similarly important to better understand the travel habits of people with SHS; more comprehensive models can be established by integrating relevant factors. Lastly, SHS domains and symptoms are closely associated with people’s lifestyles. More in-depth investigations should be undertaken to determine ways to improve the health of people with SHS by engaging with certain tourism activities and products. Doing so can help tourists escape their daily routines and improve their well-being.
